# Morbidity Burdens Attributable to Various Illnesses and Injuries Among Deployed Active and Reserve Component Service Members of the U.S. Armed Forces, 2024

**Published:** 2025-09-20

Each year, *MSMR* estimates illness and injury-related morbidity and health care burdens on the U.S. Armed Forces and the Military Health System (MHS), and this report updates previous analyses of these burden distributions among active and reserve component service members in deployed settings. While deployed service members are primarily selected from a subset of the active component, the reserve component contributes a substantial portion of U.S. deployed forces.

This report utilizes data from the Theater Medical Data Store (TMDS), which documents service members' inpatient and outpatient encounters while treated in an operational environment. TMDS receives medical data from Theater Medical Information Program-Joint (TMIP-J) applications, including AHLTA-Theater, TMIP-Composite Health Care System Cache, Mobile Computing Capability, Maritime Medical Modules, and the U.S. Transportation Command Regulating and Command and Control Evacuation System (TRAC2ES). ^1^

The health encounters of service members deployed to 2 specific theaters of operation, U.S. Central Command (CENT-COM) and U.S. Africa Command (AFRI-COM), are the subject of this report. While U.S. service members are deployed to all the geographic combatant commands, the largest concentrations without access to permanent medical facilities are in the CENTCOM and AFRICOM areas of operation. ^2^ While this report focuses on medical encounters of service members treated in CENTCOM and AFRICOM operational environments during the 2024 calendar year, future reports may incorporate other combatant commands as circumstances dictate and data become available.

## Methods

The surveillance population included all individuals who served in the active or reserve components of the U.S. Army, Navy, Air Force, Marine Corps, or Space Force with health care encounters captured in the TMDS during the surveillance period. Analysis was restricted to encounters where the theater of care specified was CENTCOM or AFRICOM, or where the name of the theater of operation was missing or null; by default, this excluded encounters in the U.S. Northern Command (NORTHCOM), U.S. European Command (EUCOM), U.S. Indo-Pacific Command (INDOPACOM), or U.S. Southern Command (SOUTHCOM) theaters of operations. In addition, TMDS-recorded medical encounters where the data source was identified as Shipboard Automated Medical System, or where the military treatment facility descriptor indicated that care was provided aboard ship, were excluded from this analysis. Encounters from aeromedical staging facilities outside of CENTCOM or AFRICOM were also excluded.

Morbidity burdens attributable to various conditions were estimated by diagnosis distribution according to the 17 traditional categories of the International Classification of Diseases (ICD) system, with an 18th category for COVID-19. Extended ICD-10 (10th Revision) code groupings were also reviewed for the most common diagnoses. The TMDS has not fully transitioned to ICD-10 codes, so some ICD-9 (9th Revision) codes were included. Primary diagnoses that did not correspond to an ICD-9 or ICD-10 code are not reported in this health care burden analysis.

**What are the new findings?**

Musculoskeletal disorders, in combination with administrative and other health services (ICD-10 ‘Z’ codes), accounted for more than half of the total medical encounters in 2024 among service members deployed to the U.S. Central Command (CENTCOM) or Africa Command (AFRICOM). Lower back pain accounted for the most frequent musculoskeletal condition among male and female service members deployed to CENTCOM and AFRICOM.

**What is the impact on readiness and force health protection?**

Thorough examination of the most common causes of injury and illness during deployment can assist senior leaders in the development and implementation of strategies to reduce preventable medical issues, enhance force readiness, and ensure fighting strength.

## Results

A total of 191,579 medical encounters occurred among 52,066 individuals deployed to Southwest Asia, the Middle East, and Africa in 2024. Of those 191,579 total medical encounters documented in 2024 among deployed service members, 227 (0.1%) were recorded as hospitalizations. Most medical encounters (n=146,384, 76.4%), individuals affected (n=42,344, 81.3%), and hospitalizations (n=181, 79.7%) occurred among male service members.

In 2024, the largest percentages of medical encounters among deployed service members were coded as musculoskeletal system / connective tissue disorders, followed by administrative and other health services (i.e., ‘Z’ codes, including factors influencing health status and health service contact) **(Figure)** . The most common diagnosis within the musculoskeletal system / connective tissue disorders group was for unspecified lower back pain (ICD-10 codes beginning with M545) **(Table)** . The percentage of total medical encounters attributed to other health services decreased from 32.1% in 2020 to 22.8% in 2024. COVID-19 accounted for only 0.3% of deployed service members' total medical encounters in 2024 **(Figure)** .

**FIGURE:**
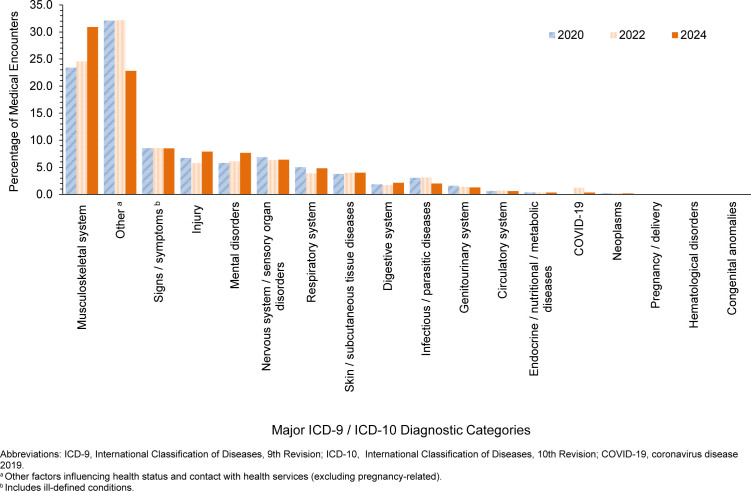
Major ICD-9 / ICD-10 Diagnostic Categories of In-Theater Medical Encounters, Active Component, U.S. Armed Forces, 2020, 2022 and 2024

**TABLE:** Most Frequent ICD-10 Diagnostic Codes for In-Theater Medical Encounters by Sex, Active Component, U.S. Armed Forces, 2024

Total				Male				Female			
ICD-10 Code ^a^	ICD-10 Code Description	No.	%	ICD-10 Code ^a^	ICD-10 Code Description	No.	%	ICD-10 Code ^a^	ICD-10 Code Description	No.	%
Z029	Encounter for administrative examinations, unspecified	9,900	5.2	Z029	Encounter for administrative examinations, unspecified	7,283	5.0	Z029	Encounter for administrative examinations, unspecified	2,617	5.8
M5450	Low back pain, unspecified	8,851	4.6	M5450	Low back pain, unspecified	7,055	4.8	M5450	Low back pain, unspecified	1,796	4.0
Z5682	Military deployment status	5,709	3.0	Z5682	Military deployment status	4,560	3.1	Z5682	Military deployment status	1,149	2.6
M25511	Pain in right shoulder	4,238	2.2	M25511	Pain in right shoulder	3,634	2.5	J069	Acute upper respiratory infection, unspecified	965	2.2
M25561	Pain in right knee	3,829	2.0	M25512	Pain in left shoulder	3,154	2.2	M25561	Pain in right knee	902	2.0
J069	Acute upper respiratory infection, unspecified	3,762	2.0	M25561	Pain in right knee	2,927	2.0	M542	Cervicalgia	791	1.8
M25512	Pain in left shoulder	3,647	1.9	M25562	Pain in left knee	2,805	1.9	M25562	Pain in left knee	724	1.6
M25562	Pain in left knee	3,529	1.9	J069	Acute upper respiratory infection, unspecified	2,797	1.9	F439	Reaction to severe stress, unspecified	662	1.5
M542	Cervicalgia	3,314	1.7	M542	Cervicalgia	2,523	1.7	Z0289	Encounter for other administrative examinations	612	1.4
Z23	Encounter for immunization	2,794	1.5	Z9182	Personal history of military deployment	2,354	1.6	M25511	Pain in right shoulder	604	1.3
Z9182	Personal history of military deployment	2,751	1.4	Z23	Encounter for immunization	2,256	1.5	M25551	Pain in right hip	555	1.2
M545	Low back pain	2,591	1.4	M545	Low back pain	2,240	1.5	F4323	Adjustment disorder with mixed anxiety and depressed mood	540	1.2
Z0289	Encounter for other administrative examinations	2,565	1.3	L731	Pseudofolliculitis barbae	2,057	1.4	Z23	Encounter for immunization	538	1.2
R197	Diarrhea, unspecified	2,205	1.2	Z0289	Encounter for other administrative examination	1,953	1.3	Z7189	Other specified counseling	525	1.2
F439	Reaction to severe stress, unspecified	2,146	1.1	R197	Diarrhea, unspecified	1,739	1.2	M25512	Pain in left shoulder	493	1.1
L731	Pseudofolliculitis barbae	2,060	1.1	F439	Reaction to severe stress, unspecified	1,484	1.0	R197	Diarrhea, unspecified	466	1.0
Z7189	Other specified counseling	1,883	1.0	Z7189	Other specified counseling	1,358	0.9	M25552	Pain in left hip	448	1.0
F4323	Adjustment disorder with mixed anxiety and depressed mood	1,820	1.0	G4726	Circadian rhythm sleep disorder, shift work type	1,306	0.9	Z719	Counseling, unspecified	446	1.0
J00	Acute nasopharyngitis [common cold]	1,577	0.8	F4323	Adjustment disorder with mixed anxiety and depressed mood	1,280	0.9	Z733	Stress, not elsewhere classified	406	0.9
M25571	Pain in right ankle, joints of right foot	1,537	0.8	J00	Acute nasopharyngitis [common cold]	1,173	0.8	J00	Acute nasopharyngitis [common cold]	404	0.9
M25551	Pain in right hip	1,498	0.8	M25572	Pain in left ankle, joints of left foot	1,146	0.8	M5459	Other low back pain	404	0.9
G4726	Circadian rhythm sleep disorder, shift work type	1,478	0.8	M25571	Pain in right ankle, joints of right foot	1,143	0.8	Z9182	Personal history of military deployment	397	0.9
Z719	Counseling, unspecified	1,459	0.8	Z719	Counseling, unspecified	1,013	0.7	M25571	Pain in right ankle, joints of right foot	394	0.9
M25572	Pain in left ankle, joints of left foot	1,445	0.8	M5459	Other low back pain	1,001	0.7	R519	Headache, unspecified	377	0.8
M5459	Other low back pain	1,405	0.7	R519	Headache, unspecified	967	0.7	M545	Low back pain	351	0.8
R519	Headache, unspecified	1,344	0.7	R21	Rash and other non-specific skin eruption	961	0.7	R21	Rash and other non-specific skin eruption	343	0.8
R21	Rash and other non-specific skin eruption	1,304	0.7	Z760	Encounter for issue of repeat prescription	958	0.7	F419	Anxiety disorder, unspecified	336	0.7
Z760	Encounter for issue of repeat prescription	1,279	0.7	M549	Dorsalgia, unspecified	956	0.7	Z760	Encounter for issue of repeat prescription	321	0.7
M549	Dorsalgia, unspecified	1,246	0.7	M25551	Pain in right hip	943	0.6	F5102	Adjustment insomnia	303	0.7
M25552	Pain in left hip	1,143	0.6	M546	Pain in thoracic spine	899	0.6	R109	Unspecified abdominal pain	302	0.7
F5102	Adjustment insomnia	1,121	0.6	F5102	Adjustment insomnia	818	0.6	M25572	Pain in left ankle, joints of left foot	299	0.7
M546	Pain in thoracic spine	1,111	0.6	G4729	Other circadian rhythm sleep disorder	810	0.6	M549	Dorsalgia, unspecified	290	0.6
Z733	Stress, not elsewhere classified	1,109	0.6	Z733	Stress, not elsewhere classified	703	0.5	Z658	Other specified problems related to psychological circumstances	249	0.6
F419	Anxiety disorder, unspecified	915	0.5	G4700	Insomnia, unspecified	698	0.5	M79671	Pain in right foot	239	0.5
G4729	Other circadian rhythm sleep disorder	915	0.5	M25552	Pain in left hip	695	0.5	M722	Plantar fascial fibromatosis	227	0.5
R109	Unspecified abdominal pain	882	0.5	Z5739	Occupational exposure to other air contaminants	676	0.5	M25531	Pain in right wrist	225	0.5
G4700	Insomnia, unspecified	851	0.4	I10	Essential (primary) hypertension	627	0.4	J029	Acute pharyngitis, unspecified	220	0.5
M722	Plantar fascial fibromatosis	821	0.4	F4320	Adjustment disorder, unspecified	598	0.4	N760	Acute vaginitis	219	0.5
Z5739	Occupational exposure to other air contaminants	808	0.4	M722	Plantar fascial fibromatosis	594	0.4	M546	Pain in thoracic spine	212	0.5
Z658	Other specified problems related to psychological circumstances	793	0.4	R109	Unspecified abdominal pain	580	0.4	F4310	Post-traumatic stress disorder, unspecified	210	0.5
M79671	Pain in right foot	792	0.4	F419	Anxiety disorder, unspecified	579	0.4	F4322	Adjustment disorder with anxiety	209	0.5

Abbreviations: ICD-10, International Classification of Diseases, 10th Revision; No., number; ICD-9, International Classification of Diseases, 9th Revision; TMDS, Theater Medical Data Store.

a Some ICD-9 codes still appear in TMDS. While medical encounters documented with ICD-9 codes were included in the overall analysis for major diagnostic category analysis, the summary of these codes are excluded from this table.

The percentages of in-theater medical encounters attributed to musculoskeletal system disorders increased from 2020 (23.4%) to 2024 (30.9%) **(Figure)** . Unspecified lower back pain (M5450) was the most frequent ICD-10 diagnostic code for musculoskeletal encounters among both men and women **(Table)** . The second-most frequent ICD-10 diagnostic code for musculoskeletal encounters among male service members was pain in the right shoulder (M25511), while for female service members it was pain in the right knee (M25561) **(Table)** .

The percentages of in-theater medical encounters attributed to mental health disorders increased slightly during the surveillance period, from 5.8% in 2020 to 7.7% in 2024 **(Figure)** . Unspecified reaction to severe stress (F439) accounted for the most frequent mental health disorder diagnoses, with a slightly higher percentage of intheater encounters for this disorder among women (1.5%) than men (1.0%) **(Table)** .

## Discussion

As in prior annual reports of illness and injury-related morbidity and care burdens in deployed settings, musculoskeletal dis-orders, in combination with administrative and other health services, accounted for more than half of the total medical encounters in theater. In prior reports during the surveillance period, encounters for COVID-19 screening contributed to an increase in encounters for administrative and other health services, as this specific Z code (Z1152) accounted for almost 5% of all in-theater medical encounters in 2022. ^3^

This report documents an increased percentage of in-theater medical encounters for musculoskeletal disorders, consistent with the 2020-2024 increased rate of in-garrison ambulatory encounters for musculoskeletal disorders. The percentage of total ambulatory encounters attributed to musculoskeletal disorders in garrison (28.1%) was similar to the percentage observed in theater (30.9%). ^4^ No absolute rate comparisons can be made due to the lack of in-theater denominator (person-time) data.

Some conditions, including diabetes, pregnancy, or congenital anomalies, often preclude service member deployment. Due to medical pre-screening, service members who are deployed demonstrate a lower rate of medical conditions that could interfere with deployment operations than their non-deployed counterparts. Deployed service members are also less likely to require medical care for pre-screened conditions.

When interpreting these results and analyses, several limitations of these data should be considered. Not all medical encounters in theaters of operations are recorded in the TMDS. Some care by intheater medical personnel occurs at small, remote, or austere locations where electronic documentation of diagnosis and treatment is infeasible, and some emergency medical care for stabilization of combat-injured service members prior to evacuation may not be routinely captured in the TMDS. Due to the exigencies of deployment settings that can complicate accurate data reporting or transmission, this report may under-estimate the true burden of health care in the areas of operations assessed.

In any review that relies on ICD coding, some diagnosis misclassification should be expected due to coding errors within the electronic health record. Although the aggregated distributions of illnesses and injuries presented in this report are compatible with assessments derived from other examinations of morbidity in military populations (both deployed and non-deployed), instances of highly unlikely diagnostic codes for a deployed population have been observed. This misclassification bias is likely minor and non-differential.

Because this report only includes medical encounters from CENTCOM and AFRICOM, it does not describe any medical encounters from the recent deployment of troops to EUCOM, INDOPACOM, and SOUTHCOM. Each area of operation is unique, with vastly different medical assets, medical evacuation capabilities, and deployed service member populations. Consequently, the results from CENTCOM or AFRICOM may not be generalizable to other combatant commands.
